# Edible Bird’s Nest attenuates high fat diet-induced oxidative stress and inflammation via regulation of hepatic antioxidant and inflammatory genes

**DOI:** 10.1186/s12906-015-0843-9

**Published:** 2015-09-04

**Authors:** Zhang Yida, Mustapha Umar Imam, Maznah Ismail, Zhiping Hou, Maizaton Atmadini Abdullah, Aini Ideris, Norharina Ismail

**Affiliations:** Laboratory of Molecular Biomedicine, Institute of Bioscience, Universiti Putra Malaysia, 43400 Serdang, Selangor Malaysia; Cardiology Department, Affiliated Hospital of Chengde Medical University, 067000 Chengde, Hebei China; Department of Nutrition and Dietetics, Faculty of Medicine and Health Sciences, Universiti Putra Malaysia, 43400 Serdang, Selangor Malaysia; Department of Pathology, Faculty of Medicine and Health Sciences, Universiti Putra Malaysia, 43400 UPM Serdang, Selangor Malaysia; Faculty of Veterinary Medicine, Universiti Putra Malaysia, 43400 Serdang, Selangor Malaysia

**Keywords:** Antioxidants, Edible bird’s nest, High fat diet, Inflammation, Oxidative stress

## Abstract

**Background:**

Edible Bird’s nest (EBN) is an antioxidant-rich supplement that is popular in many parts of Asia. Its antioxidant and anti-inflammatory properties have been reported using in vitro system. This paper aimed to determine the antioxidant and anti-inflammatory effects of EBN in in high fat diet induced rats model.

**Methods:**

We evaluate if those properties can be translated in rats. High fat diet (HFD) was fed to rats for 12 weeks to determine its effects on oxidative stress and inflammation, and compared with HFD + Simvastatin and HFD + EBN (2.5 or 20 %). Weights were measured weekly, while serum and hepatic markers of oxidative stress (total antioxidant status and TBARS) and inflammation (interleukin 6 [IL-6], C-reactive protein [CRP] and tumor necrosis factor alpha [TNF-α]) were determined at the end of the intervention. In addition, transcriptional changes in hepatic antioxidant (superoxide dismutase, glutathione reductase, glutathione peroxidase) and inflammation (C-reactive protein, chemokine [C-C] motif 2, nuclear factor kappa beta 1 and tumor necrosis factor alpha) genes were evaluated.

**Results:**

The results showed increases in oxidative stress (raised TBARS and lowered total antioxidant status) and inflammatory markers (raised CRP, IL-6 and TNF-α) in HFD induced rats with corresponding attenuation of antioxidant gene expression and potentiation of inflammatory gene expression. EBN on the other hand attenuated the HFD-induced inflammation and oxidative stress and produced overall better outcomes in comparison with simvastatin.

**Conclusions:**

In aggregate, the results support the evidence-based utilization of EBN as a supplement for preventing obesity-related inflammation and oxidative stress in rats. These promising results can open up opportunities for translating the benefits of EBN to humans.

## Background

The contribution of inflammation to many disease processes is well established. In fact, many chronic diseases have been associated with low grade inflammation [1]. Cardiometabolic diseases have been growing steadily over the years and their burden is expected to rise significantly in the near future if no urgent action is taken. Despite advances in medical healthcare, the currently available strategies for the management of cardiometabolic diseases have not been completely effective in controlling the burden of the diseases. Moreover, side effects are limiting the use of some of these therapies, and hence, the search for newer alternatives. Additionally, links between diet and chronic disease development are now strongly acknowledged, and this new understanding has widened the search for alternative therapies to closely consider dietary methods of managing cardiometabolic diseases [2].

Edible bird’s nest (EBN) is a traditional supplement consumed for its perceived health-promoting properties, especially among Asians. However, there is lack of scientific research to support the evidence-based use of EBN as a supplement. Recent reports indicate that it possesses antioxidative and anti-inflammatory effects [3–5] but the precise mechanisms underlying such effects are not completely understood. Moreover, these reports are from in vitro systems, which sometimes do not reflect what happens in in vivo systems. Furthermore, the bioactivity of foods with multiple constituents is often attributed to their synergistic effects [6]. This is true of EBN, which has many different constituents including proteins, carbohydrates, fatty acids and other minerals [7]. Additionally, we have recently reported that sialic acid is a major protein constituent of EBN (11 % of EBN), and may in fact be contributing significantly to its bioactivity [8]. However, the contribution of its multiple bioactives towards any of its effects cannot be ruled out in view of the importance of food synergy in foods with multiple constituents like EBN [6]. Thus, in view of the lack of information on the effects of EBN on in vivo oxidative stress and inflammation and the underlying mechanisms involved, we tested the hypothesis that EBN regulated hepatic antioxidant and anti-inflammatory genes in rats as the basis for its antioxidative and anti-inflammatory properties.

## Methods

### Materials

C-reactive protein (CRP), interleukin 6 (IL6) and tumor necrosis factor alpha (Tnf-α) ELISA kits were purchased from Elabscience Biotechnology Co., Ltd (Wuhan, China), while 1,1,3,3-tetramethoxypropane (TMP), thiobarbituric acid, potassium persulphate (K_2_S_2_O_8_), 2,2’-azino-bis [3-ethylbenzothiazoline-6-sulphonic acid] (ABTS) reagent, trolox standard and trichloroacetic acid were purchased from Sigma Aldrich (St. Loius, MO, USA). RNA extraction kit was from RBC Bioscience Corp. (Taipei, Taiwan) and GenomeLab™ GeXP Start Kit was from Beckman Coulter Inc (Miami, FL, USA). Simvastatin, RCL2 solution and lipid profile kits were from Pfizer (New York, NY, USA), Alphelys (Toulouse, France) and Randox Laboratories Ltd (Crumlin, County Antrim, UK), respectively. Cholesterol and Cholic acid were purchased from Amresco (Solon, OH, USA) and Santa Cruz Biotechnology (Santa Cruz, CA, USA), respectively. Standard rat pellet was from Specialty feeds (Glen Forrest, WA, USA), while palm oil was supplied by Yee Lee Edible oils Sdn. Bhd. (Perak, Malaysia). All other solvents were of analytical grade and purchased from Merck (Darmstadt, Germany). EBN supplied by Blossom View Sdn. Bhd (Terrengganu, Malaysia) was cleaned under tap water for 5 mins, dried at room temperature and ground into powder manually using mortar and pestle before incorporating it into rat pellet.

### Animal study

Permission for the use of animals was sought from the Animal Care and Use Committee (ACUC) of the Faculty of Medicine and Health Sciences, Universiti Putra Malaysia (Project approval number: UPM/IACUC/AUP-R011/2014), and animals were handled as according to standard guidelines. Sprague dawley rats (10-week old, 230–280 g, *n* = 30) were maintained under controlled conditions (25 ± 2 °C, 12/12 h light/dark cycle) and after 2 weeks of acclimatization rats were fed HFD containing 4.5 % cholesterol and 0.5 % cholic acid with or without treatment using Simvastatin or EBN (Table 1), except the normal group (*n* = 6). Intervention lasted for another 12 weeks, after which rats were sacrificed and their organs harvested for further studies. During the intervention, food intake was calculated by subtracting the left-over food from what was added the previous day, and weight was recorded weekly. At the end of the intervention, blood samples and liver tissues were collected for further tests.

**Table 1:** Food composition of rat groups and intake

Animal group	Normal pellet (%)	Cholesterol/cholic acid (%)	Palm oil (%)	Starch (%)	Others	Food intake (Kcal/kg/day)	Initial weight (g)	Final weight (g)
Normal	100					215.5 ± 33.5^a^	260.4 ± 10.7^a^	384 ± 22.9^a^
High fat diet	65	5	20	10		215.0 ± 37.5^a^	262.6 ± 17.7^a^	395.2 ± 16.8^a^
High fat diet + Simvastatin	65	5	20	10	Simvastatin (10 mg/kg)	215.7 ± 36.6^a^	267.7 ± 21^a^	375.7 ± 53.4^a^
High fat diet + 20 % EBN	45	5	20	10	20 % EBN	216.1 ± 36.8^a^	261.7 ± 15.4^a^	380.7 ± 25.6^a^
High fat diet + 2.5 % EBN	62.5	5	20	10	2.5 % EBN	216.5 ± 35.8^a^	257 ± 20.1^a^	368 ± 29.3^a^

EBN: edible bird’s nest

Similar superscript letters (a) in each column indicate no statistically significant difference (p<0.05)

### Liver Thiobarbituric acid reactive species (TBARS)

Liver tissue TBARS was measured as reported previously with some modifications [9]. Briefly, liver tissue (80 mg) was homogenized in 250 uL of 0.25Hcl and mixed with 250 uL of 0.375 % thiobarbituric acid and 250 uL of 15 % tricholoroacetic acid. Then, the mixture was incubated at 100 °C for 10 min and cooled before centrifuging at 3000 rpm for 15 min. Finally, absorbance of 100 uL of each sample and standard (TMP) were measured on BioTeK Synergy H1 Hybrid Reader (BioTek Instruments Inc., Winooski, VT, USA) at 532 nm, and the results expressed as uM MDA/mg tissue.

### Serum total Antioxidant status

ABTS radical scavenging activity was used as a marker of total antioxidant status [10]. Briefly, 6.62 mg of potassium persulphate, K_2_S_2_O_8_ was dissolved in 10 mL of distilled water to prepare a solution of 2.45 mM. Then, 7 mM ABTS was prepared by dissolving 38.4 mg in 10 mL distilled water. The two solutions were mixed and incubated in the dark for 16 h prior to use, and diluted with distilled water until a spectrophotometric absorbance of 0.700 ± 0.005 at 735 nm was obtained. Serum samples (100 μL) were reacted with 900 μL of the diluted ABTS solution and vortexed. The absorbance was read at 734 nm. ABTS radical cation scavenging activity was calculated as the percentage reduction in absorbance.

### Serum C-reactive protein, Interleukin-6 and Tumor necrosis factor-alpha

Serum from blood collected in plain tubes was used for measurements of CRP, IL6 and Tnf-α using the respective ELISA kits according to the manufacturers’ instructions. Absorbances were read on BioTeK Synergy H1 Hybrid Reader (BioTek Instruments Inc., Winooski, VT, USA) at the 450 nm. The results were finally expressed as fold change in comparison to normal group:

Fold change = absorbance for the intervention group/absorbance for the normal group

### Gene expression study

Primers for the gene expression study (Table 2) were designed using the *Rattus norvegicus* gene sequences from the National Center for Biotechnology Information website (http://www.ncbi.nlm.nih.gov/nucleotide/), and tagged with an 18-nucleotide universal forward and 19-nucleotide universal reverse sequence, respectively. Primers were supplied by Integrated DNA Technologies (Singapore) and reconstituted in RNAse free water. Extracted hepatic RNA (20 ng) was reverse transcribed and used for PCR according to the GenomeLab™ GeXP Start Kit protocol (Beckman Coulter, USA), using the conditions stated on Table 2. PCR products (1 uL) were analyzed on GeXP genomelab genetic analysis system (Beckman Coulter, Inc, Miami, FL, USA) after mixing with sample loading solution and DNA size standard 400 as recommended by the manufacturer. Results were analyzed with the Fragment Analysis module of the GeXP system software and normalized on the eXpress Profiler software.

**Table 2:** Names, accession number and primer sequences used in the study

Name	Left sequence	Right sequence
nfkb1	AGGTGACACTATAGAATACACTCCATATTTAATGCAGA	GTACGACTCACTATAGGGAGAAATCCTCTCTGTTTCG
B2m^a^	AGGTGACACTATAGAATAATGCTTGCAGAGTTAAACA	GTACGACTCACTATAGGGATGCATAAAATATTTAAGGTAAGA
Hprt1^a,b^	AGGTGACACTATAGAATATCCTCATGGACTGATTATG	GTACGACTCACTATAGGGACTGGTCATTACAGTAGCTCTT
Rpl13a^a^	AGGTGACACTATAGAATAATGGGATCCCTCCAC	GTACGACTCACTATAGGGAATTTTCTTCTCCACATTCTT
Kan(r)^c^		
Sod1	AGGTGACACTATAGAATAATATGGGGACAATACACAA	GTACGACTCACTATAGGGATCCAACATGCCTCTCT
Sod2	AGGTGACACTATAGAATACAGGTTGCTCTTCAGC	GTACGACTCACTATAGGGAAACTCTCCTTTGGGTTCT
Tnf	AGGTGACACTATAGAATACCCAACAAGGAGGAGA	GTACGACTCACTATAGGGATGGTGGTTTGCTACGA
CcL2	AGGTGACACTATAGAATAATAAAATTGCATCAACCCTA	GTACGACTCACTATAGGGAATCACATTCCAAATCACACT
IL6	AGGTGACACTATAGAATAATTGTATGAACAGCGATGA	GTACGACTCACTATAGGGAAGTTGTTCTTCACAAACTCC
Gpx1	AGGTGACACTATAGAATATTGAGAAGTTCCTGGTAGGT	GTACGACTCACTATAGGGATTTTCTGGAAATCAGGTGT
IL10	AGGTGACACTATAGAATAAGGACTTTAAGGGTTACTTG	GTACGACTCACTATAGGGAGAAGATGTCAAACTCATTCA
Gsr	AGGTGACACTATAGAATAAATAAACTGGGGATTCAGAC	GTACGACTCACTATAGGGAAGTAGATTTTCACATTGTCTTTG
CRP	AGGTGACACTATAGAATACTAAACAGGCCTTCGTATT	GTACGACTCACTATAGGGACAAGCCAAAGCTCTACAAT

^a^Housekeeping genes. ^b^Normalization gene. Underlined sequences are left and right universal left and right sequences (tags). ^c^internal control supplied by Beckman Coulter Inc (Miami, FL, USA) as part of the GeXP kit. RT conditions were: 48 °C for 1 min; 37 °C for 5 min; 42 °C for 60 min; 95 °C for 5 min, then hold at 4 °C. PCR conditions were initial denaturation at 95 °C for 10 min, followed by two-step cycles of 94 °C for 30 s and 55 °C for 30 s, ending in a single extension cycle of 68 °C for 1 min. B2m: Beta-2-Microglobulin; Ccl2: Chemokine (C-C Motif) Ligand 2; CRP: C-reactive protein; Gpx1: glutathione peroxidase 1; Gsr: glutathione reductase; Hprt1: Hypoxanthine Phosphoribosyltransferase 1; IL6: interleukin 6;  IL10: interleukin 10; KanR: Kanamycin resistant; nfkb1: nuclear factor Kappa B 1; Rpl13a: Ribosomal Protein L13a; Sod:superoxide dismutase; Tnf: tumor necrosis factor

#### Data analysis

The means ± standard deviation (*n* = 6) of the groups were used for the analyses. One-way analysis of variance (ANOVA) was performed using SPSS 17.0 software (SPSS Inc., Chicago, IL, USA) to assess the level of significance of differences between means with a cutoff of *p* < 0.05.

## Results and discussions

### EBN attenuated high fat diet-induced oxidative stress

**Fig. 1:**
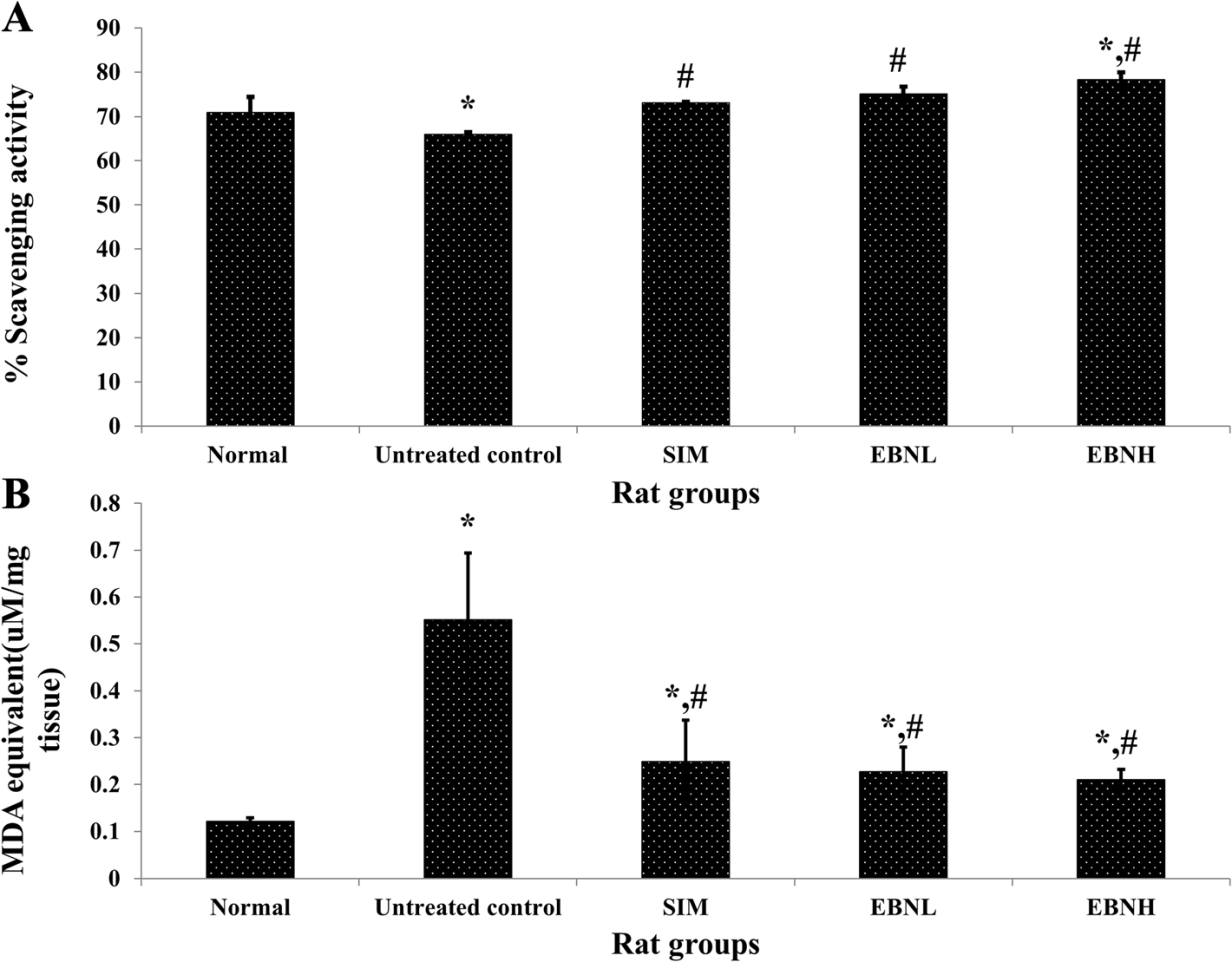
Effect of edible bird’s nest (EBN) on **a**) serum antioxidant status (expressed as % scavenging activity in relation to blank) and **b**) liver thiobarbutric acid reactive species (expressed as malondialdehyde [MDA] equivalent) after 12 weeks intervention. The normal group received standard rat chow, while the other groups received HFD containing 4.5 % cholesterol and 0.5 % cholic acid (untreated control group), HFD containing 4.5 % cholesterol and 0.5 % cholic acid + 20 mg/kg/day Simvastatin (SIM), HFD containing 4.5 % cholesterol and 0.5 % cholic acid + 2.5 % EBN (EBNL, EBN low) or HFD containing 4.5 % cholesterol and 0.5 % cholic acid + 20 % EBN (EBNH, EBN high). * indicates significant difference in comparison with the normal group (*p* < 0.05). # indicates significant difference in comparison with the untreated control group (*p* < 0.05)

High fat diet-induced weight gain is often accompanied by oxidative stress [11, 12]. In this study, high fat diet increased weight gain and induced oxidative stress, which were attenuated by EBN feeding (Table 1 & Fig. 1). The results indicate that while high fat diet promoted oxidative stress relative to normal group, EBN significantly attenuated such a response, although no significant differences were observed between the EBN groups. Moreover, increased weight has been associated with low grade inflammation and other cardiometabolic health problems [13]. The level of TBARS in the Simvastatin group was also lower than in the untreated control group, but not significantly so. Furthermore, antioxidant status in the different groups indicated that high fat diet reduced antioxidants in comparison with the normal group, while EBN prevented such an effect. Simvastatin was similarly able to prevent reduction in antioxidant status. The ability of EBN to reduce oxidative stress has been ascribed to its antioxidant content, as demonstrated in our recent publication where EBN was shown to possess potent antioxidant abilities that contributed towards lowering of oxidative sterss markers [4]. This is similar to what has been reported on the ability of antixidants to scavenge free radicals thereby lowering oxidative stress [14]. Conversely, the weight and oxidative stress modulatory effects of Simvastatin in rats have been reported previously [15].

### EBN attenuated high fat diet induced inflammation

**Fig. 2:**
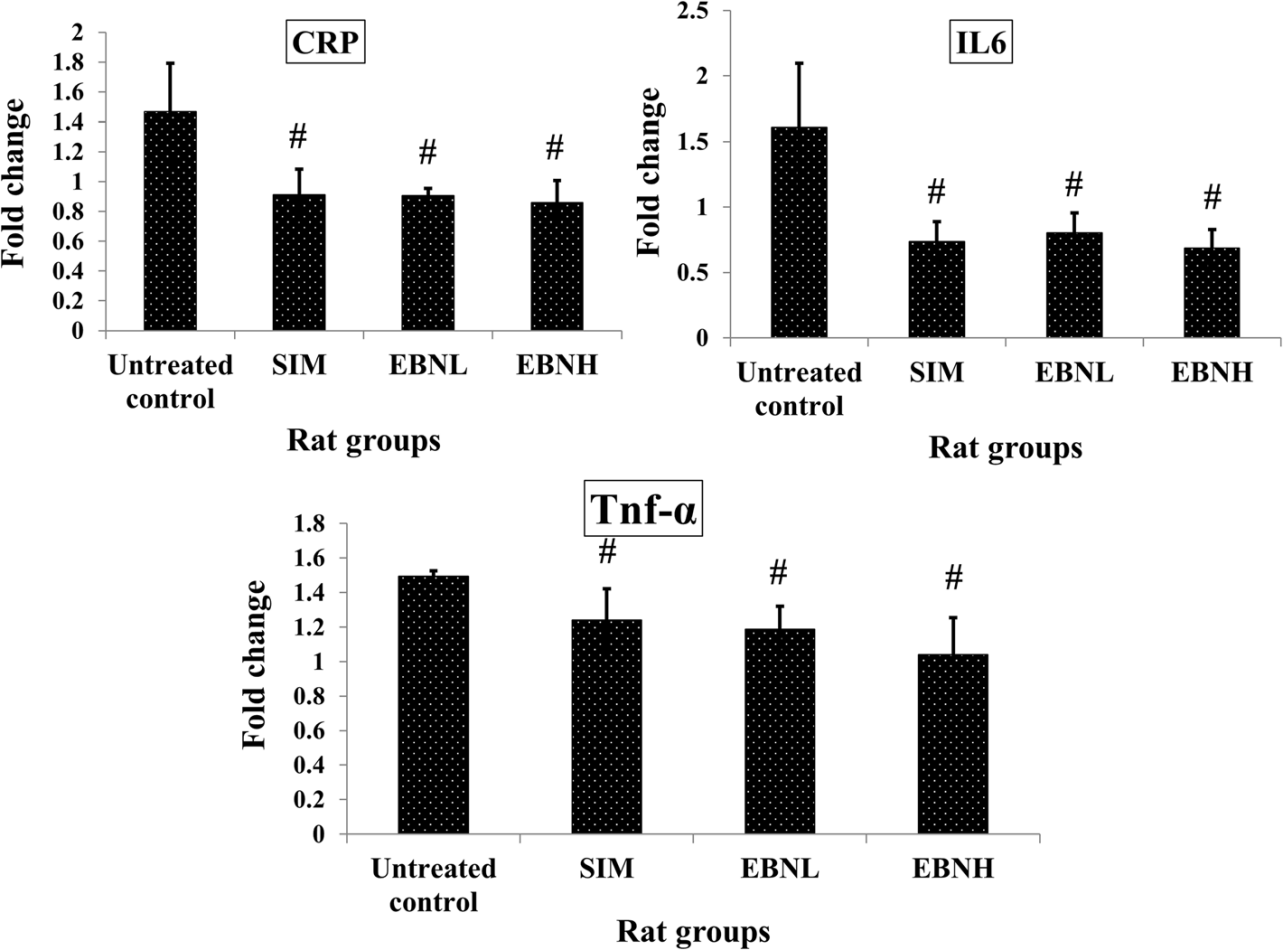
Effect of edible bird’s nest (EBN) on serum markers of inflammation after 12 weeks intervention. Groupings are the same as Fig. 1. # indicates significant difference in comparison with the untreated control group (*p* < 0.05). CRP: C-reactive protein; IL6: interleukin 6; TNF-α: tumor necrosis factor alpha

In the present study, EBN also attenuated EBN-induced inflammatory response after 12 weeks of intervention (Fig. 2). C-reactive protein (CRP), interleukin (IL) 6 and tumor necrosis factor alpha (Tnf-α) were lower in the EBN and Simvastatin groups. These results are in agreement with previous findings on the anti-inflammatory effects of EBN and Simvastatin [16]. Elevated CRP and IL6 have been reported as important markers of the inflammatory response [17], while Tnf-α is involved in the mediation of a number of processes including the inflammatory process [18]. Moreover, decreases in serum levels of these markers (CRP, IL6 and Tnf-α) has been associated with reduced inflammation [17, 18], and therefore, the present results suggest that EBN has anti-inflammatory effects in in vivo systems. It will be recalled that the anti-inflammatory effects of EBN have thus far been reported using in vitro system [3], and our report provides more insight into such effects.

### EBN regulated hepatic antioxidant and inflammatory genes

**Fig. 3:**
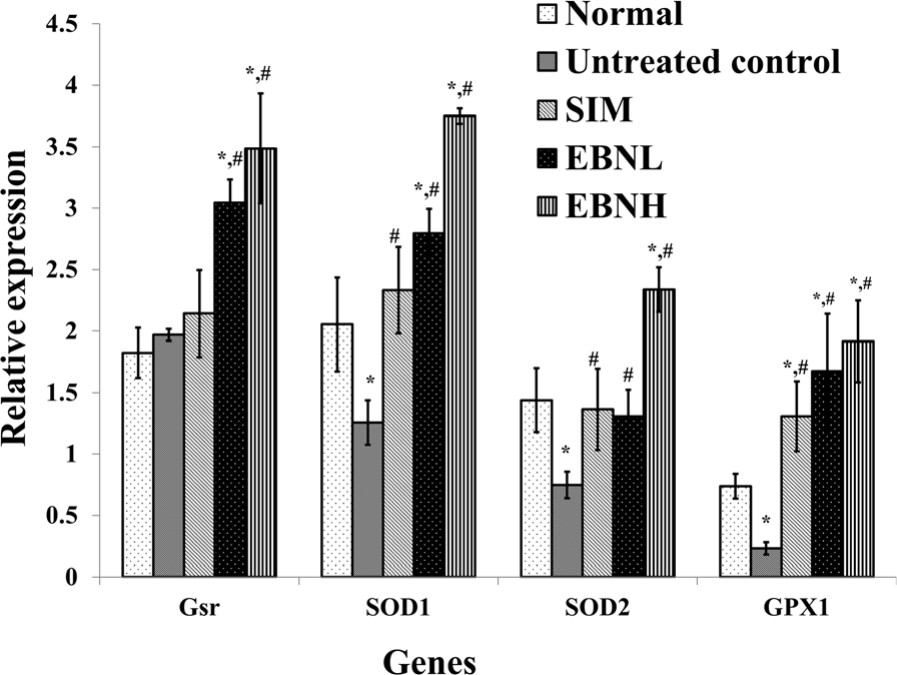
Effect of edible bird’s nest (EBN) on hepatic mRNA levels of antioxidant genes after 12 weeks intervention. Groupings are the same as Fig. 1. * indicates significant difference in comparison with the normal group (*p* < 0.05). # indicates significant difference in comparison with the untreated control group (*p* < 0.05). SOD: superoxide dismutase; Gsr: glutathione reductase; Gpx: Glutathione peroxidase

Based on the antioxidant and anti-inflammatory effects shown by EBN, we evaluated its effects on hepatic antioxidant and inflammation-related genes (Table 1). High fat diet feeding suppressed the expression of hepatic antioxidant genes (Fig. 3; superoxide dismutase [SOD] 1 and 2, glutathione reductase [Gsr], and glutathione peroxidase [Gpx]), while EBN increased their expression. The results suggest that increased expression of antioxidants through transcriptional regulation may partly underlie the increased antioxidant status in the rats that consumed EBN. Moreover, increased expression of hepatic antioxidant genes has been associated with improved antioxidant status [19]. Also, EBN showed a dose-dependent effect on the expression of antioxidant genes but not serum total antioxidant status possibly because post-transcriptional modifications produced effects to the same degree irrespective of the degree of transcriptional changes induced. The results suggest that EBN will ultimately produce an all-or-none effect notwithstanding the transcriptional changes.

**Fig. 4:**
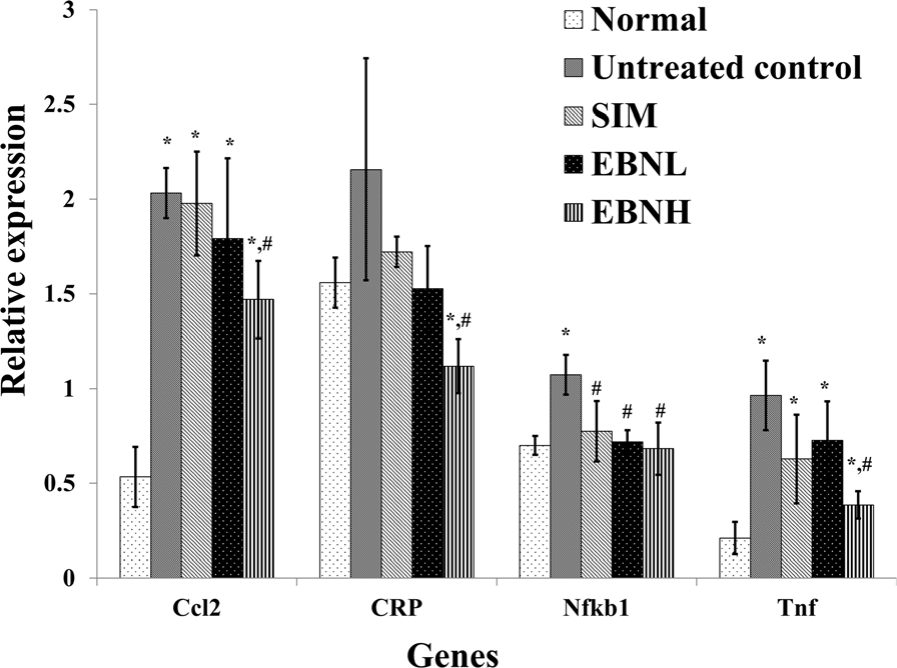
Effect of edible bird’s nest (EBN) on hepatic mRNA levels of inflammation-related genes after 12 weeks intervention. Groupings are the same as Fig. 1. * indicates significant difference in comparison with the normal group (*p* < 0.05). # indicates significant difference in comparison with the untreated control group (*p* < 0.05). Ccl2: chemokine (C-C motif) ligand 2; CRP: C-reactive protein; Nfkb1: nuclear factor kappa B1; Tnf: tumor necrosis factor

Furthermore, high fat diet feeding increased expression of inflammatory genes (Fig. 4; CRP Tnf-α, chemokine [C-C motif] ligand 2 [CCL2], and nuclear factor Kappa B [nfkb]1), while EBN prevented these high fat diet-induced changes. Increased expression of hepatic inflammatory markers and suppression of the anti-inflammatory marker suggested that high fat diet favoured an increased inflammatory response, in keeping with earlier findings [13]. EBN, on the other hand, prevented the increase in expression of hepatic inflammatory markers, which may underlie the reduced serum inflammatory markers observed in this study; CRP and Tnf-α were reduced in the serum of the EBN groups. However, just as we observed for the hepatic antioxidant genes, the dose-dependent transcriptional changes induced by EBN were not reflected by the results of the serum biomarkers. As can be recalled, these observations may be due to post-transcriptional modifications that produce an all-or-none effect. Interestingly, despite lack of effect of EBN on the expression of the IL6 gene (data not shown), we observed a change in its serum levels. The reduced serum IL6 despite lack of any transcriptional effect of EBN on the IL6 gene EBN further supports our hypothesis that EBN may have multiple targets, and that the transcriptional changes observed in this study may only partly explain the antioxidant and anti-inflammatory effects of EBN. It is clear that other mechanisms are involved in these effects, and are worth studying further.

In aggregate, the ability of EBN to prevent high fat diet-induced weight gain, oxidative stress and inflammation suggests that it may be used as supplement to prevent obesity-associated inflammation and consequent cardiometabolic problems. Already, there are indications that inflammation due to obesity may underlie the cardiovascular and other metabolic diseases that are sequelae of excessive weight gain [13], and hence EBN may be a good supplement for these problems since it can prevent inflammation, oxidative stress and weight gain. Recently, we demonstrated that the EBN used in this study is predominantly made up of proteins and its effects were likely mediated through synergistic effects of its protein bioactives including sialic acid, lactoferrin and ovotransferrin [8]. Other bioactives may be contributing to this synergistic effect, and these findings are worth evaluating further. The presence of contaminants has also been reported in EBN, which may counter any beneficial effects of EBN. Therefore, future endeavours at studying the health effects of EBN and especially its commercialization must employ stringent screening methods to ensure no toxicity from consumption of EBN.

## Conclusions

The data demonstrated that EBN attenuated high fat diet-induced oxidative stress and inflammation partly through transcriptional regulation of hepatic antioxidant and inflammation-related genes, better than Simvastatin. The results also indicated that other post-transcriptional mechanisms may be involved in antioxidant and anti-inflammatory effects of EBN in rats. It is hoped that these findings will open up opportunities for translating the benefits of EBN to humans, and are worth evaluating further.
